# Current Status and Limitations of Myocardial Infarction Large Animal Models in Cardiovascular Translational Research

**DOI:** 10.3389/fbioe.2021.673683

**Published:** 2021-04-29

**Authors:** Hye Sook Shin, Heather Hyeyoon Shin, Yasuhiro Shudo

**Affiliations:** ^1^Department of Cardiothoracic Surgery, Stanford University School of Medicine, Stanford, CA, United States; ^2^Stanford Cardiovascular Institute, Stanford University School of Medicine, Stanford, CA, United States; ^3^Department of Neurology and Neurological Sciences, Stanford University School of Medicine, Stanford, CA, United States

**Keywords:** myocardial infarction, heart failure, large animal models, large animal surgery, preclinical, translational research, review

## Abstract

Establishing an appropriate disease model that mimics the complexities of human cardiovascular disease is critical for evaluating the clinical efficacy and translation success. The multifaceted and complex nature of human ischemic heart disease is difficult to recapitulate in animal models. This difficulty is often compounded by the methodological biases introduced in animal studies. Considerable variations across animal species, modifications made in surgical procedures, and inadequate randomization, sample size calculation, blinding, and heterogeneity of animal models used often produce preclinical cardiovascular research that looks promising but is irreproducible and not translatable. Moreover, many published papers are not transparent enough for other investigators to verify the feasibility of the studies and the therapeutics’ efficacy. Unfortunately, successful translation of these innovative therapies in such a closed and biased research is difficult. This review discusses some challenges in current preclinical myocardial infarction research, focusing on the following three major inhibitors for its successful translation: Inappropriate disease model, frequent modifications to surgical procedures, and insufficient reporting transparency.

## Introduction

Cardiovascular diseases (CVDs) are devastating health problems worldwide; they accounted for 18.6 million deaths globally in 2019, which amounted to an increase of 17.1% since 2010 (Virani et al., 2021). Myocardial ischemia is the most prevalent cause of death within the spectrum of cardiovascular illnesses. Myocardial ischemia occurs when blood flow to the myocardium is obstructed by a partial or complete blockage of the coronary artery due to plaque buildup (atherosclerosis). Coronary artery narrowing and plaque rupture causes insufficient oxygen delivery to the myocardium, causing myocardial infarction (MI). The American Heart Association estimates that a new MI case is diagnosed every 40 s in the United States (Virani et al., 2021). Over the past several decades, the pathophysiological mechanisms driving these cardiovascular complications have extensively been studied in animal models, resulting in the development of numerous interventional and pharmacological treatments (Nicolini and Gherli, 2009).

Various therapeutic strategies have been proposed to mitigate the risk of myocardial infarction with cardioprotective effects in preclinical studies, but only a few have shown positive clinical study results (Bolli et al., 2004; Kloner, 2013). Ischemic remote, pre-, per-, or post-conditioning (i.e., a series of alternating intervals of brief ischemia and reperfusion) and pharmacological manipulation have been extensively studied over the last 30 years to treat acute myocardial infarction with many positive conclusions and discoveries of many pharmacological targets in preclinical settings (Heusch, 2015). However, most of the clinical outcomes remain mixed or statistically underpowered (Heusch, 2013; Kloner, 2013; Hausenloy and Yellon, 2016; Giustino and Dangas, 2017). For example, reperfusion therapy, often coupled with the administration of adjunctive therapies, has shown to reduce infarct size in animal models of acute myocardial infarction (AMI) and improve left ventricular function; however, it has failed to show similar effects in human AMI patients, potentially due to significant discrepancies between different preclinical animal models and clinical situations (Cannon, 2005; Dirksen et al., 2007; Miura and Miki, 2008; Trankle et al., 2016).

Several cardiac repair strategies have been recently developed with promising preclinical results but also with little translational success. One strategy is the direct injection of cells or biomimetic scaffolds made of polymers with cells, growth factors, or cytokines (Ungerleider and Christman, 2014). However, the grafted cells directly injected through a needle into the myocardium easily aggregate and undergo necrosis, and they are poorly localized on the myocardium of interest, thus limiting the efficacy of the therapy (Menasché, 2018). The tissue engineering using biomaterial scaffolds is limited due to their questionable immuno- or bio-compatibility and bio-functionality (Christman and Lee, 2006; Guo et al., 2020). As an alternative, scaffold-free stem cell sheet treatment has been developed with increased cell engraftment and survival on the host myocardium and promising therapeutic effects in animal studies (Shudo et al., 2011, 2013, 2014), but there are not yet many clinical studies to date (Miyagawa et al., 2017).

Despite the disagreement over the optimal cell type, cell counts, cell delivery methods, and unknown therapeutic mechanisms, stem cell therapies seem to demonstrate some degree of therapeutic improvements in terms of reduced ischemic injury size or improved left ventricular function in MI animal models in preclinical studies (Laflamme et al., 2007; Wang et al., 2009; Wolf et al., 2009; Shudo et al., 2011; Lu et al., 2012; Okura et al., 2012; Li et al., 2013; Chong et al., 2014; Zhao et al., 2014; Alestalo et al., 2015; Haller et al., 2015; Suzuki et al., 2016; Kim et al., 2017; Sharp et al., 2017; Lim et al., 2018; Crisostomo et al., 2019; Ishida et al., 2019; Romagnuolo et al., 2019; Sun et al., 2020). Nevertheless, the promising results of many preclinical studies on cell therapies have not been successfully replicated in randomized clinical trials (Janssens et al., 2006; Lunde et al., 2006; Penicka et al., 2007; Makkar et al., 2012; Perin et al., 2012; Gao et al., 2013; Quyyumi et al., 2017; Wollert et al., 2017). According to the review of articles on PubMed (preclinical) and ClinicalTrials.Gov (clinical research), no regenerative medicine was commercialized between 2008 and 2014, and only about 50 cell therapies and eight gene therapies moved onto the clinical phase, although there had been approximately 800 preclinical studies per year (Ungerleider and Christman, 2014). The frequent failure to translate the cardio-protective and regenerative therapeutics from the bench to the bedside has been attributed to the large gap between animal models and humans and inadequate preclinical study design (Bolli et al., 2004; Kloner and Rezkalla, 2004; Downey and Cohen, 2009; Hausenloy et al., 2010; Ludman et al., 2010; Heusch, 2017). There is a growing concern over the safety and efficacy of regenerative therapeutics, which many researchers have determined to be due to low internal and external validities in preclinical animal research (Ioannidis, 2005, 2016; Bracken, 2009; van der Worp et al., 2010; Hooijmans and Ritskes-Hoitinga, 2013; Steele et al., 2017; Pound and Ritskes-Hoitinga, 2018; Voelkl et al., 2018; Lüscher, 2019; Ferreira et al., 2020). This review addresses the issues prevalent in preclinical MI research, which hinder the successful therapeutic translation of promising treatment strategies. The review proceeds by discussing (1) the obstacles in building a representative animal model for MI studies, (2) factors limiting the scientific rigor in the MI study design, and (3) suggestions for improving the relevance of preclinical MI studies.

## Review

### Suitability of Animal Models for Human MI

A major hurdle in clinical translation from bench to bedside for MI therapies is the difficulty in creating a representative disease model. Modeling MI induced heart failure (HF) that resembles human cardiac conditions is challenging because human MI develops as a result of the interplay of many causes over time and is often complicated by comorbidity and polypharmacy (Pound and Ritskes-Hoitinga, 2018). A wide range of comorbid health conditions, such as epilepsy, smoking, alcoholism, cancer, diabetes, and rheumatoid arthritis, are known to remarkably affect MI fatality (Quintana et al., 2018). The incidence of HF caused by MI is often age- and gender-biased, with higher rates in men than women and in the elderly than young adults (Savarese and Lund, 2017; Virani et al., 2021). Specific racial and ethnic populations, especially minority groups, are at a considerable risk of developing MI, which may lead to death (Graham, 2015, 2016; Virani et al., 2021). However, many animal studies have failed to reflect the heterogeneity observed in the patients with MI. The animal models currently used in the laboratory settings tend to be relatively homogeneous, young, and healthy, with no genetic predisposition or underlying medical conditions (van der Worp et al., 2010; Pound and Ritskes-Hoitinga, 2018). Many preclinical studies induce MI through direct ligation of coronary artery, which does not represent the natural pathophysiology of atherosclerosis that develops over life time in humans (Getz and Reardon, 2012; Gao et al., 2016; Lee et al., 2017). Different species are used to recapitulate the pathogenesis of MI with its own advantages and disadvantages. Small animal models (rodents) are widely used in MI studies for their practical benefits, such as small body size, easy pre-/post-care, low maintenance cost, shorter generation time, and well-defined genetics. However, small animals have limitations in that their anatomy and cardiac kinetics are fundamentally different from those of humans. For example, rodent hearts function at very high heart rates (HRs), with their resting HR being more than five times higher than in humans. Their small body and organ sizes and short lifespan require expression of different genes related to action potential properties and contractile kinetics in ventricular cardiomyocytes (CMs) (Locher et al., 2009; Milani-Nejad and Janssen, 2014). For example, their ventricular CMs predominately express fast α-myosin heavy chain (MHC) (>94–100%), whereas human LV cardiomyocytes (CMs) predominately expresses slow β-MHC (>90–95%), thus resulting in differential cardiac contractile and kinetic responses to cardiac dysfunction (Milani-Nejad and Janssen, 2014). These differences in cardiac parameters may lead to different results of cell therapy experiments across different animal models. For example, Laflamme et al. (2007) observed frequent arrhythmias in non-human primates and pigs following transplantation of embryonic stem cell-derived cardiomyocytes, but not in rats, possibly because rats’ high heart rate could mask arrhythmias (Chong et al., 2014; Romagnuolo et al., 2019).

Small animals’ body and organ sizes make it even more challenging to mimic the natural pathophysiology of human atherosclerosis and thus MI. The gradual occlusion of the coronary artery can be established in animal models by using interventional operation using various materials, such as Ameroid Constrictors (Shudo et al., 2011; Potz et al., 2018; Ishida et al., 2019). However, small animals’ heart is too small to correctly identify each vasculature, which is tricky to occlude using these materials. The most feasible way to induce MI in small animals is the permanent ligation of the coronary artery using a suture loop, but the etiology is different from that naturally occurring MI in humans in this case. Even though there have been attempts to model atherosclerosis in transgenic or high fat-fed rodents, rodents rarely develop atherosclerosis in coronary arteries but readily in the aortic root probably due to their rapid heart rate and blood flow and often in the absence of complications seen in human MI patients such as thrombosis (Getz and Reardon, 2012; Gao et al., 2016; Lee et al., 2017).

Besides, small animals’ cardiac anatomy and physiology make it challenging to visualize and quantify the spatial distribution of blood flow and assess microvascular histomorphology following MI (Krueger et al., 2013; Liu et al., 2020). To overcome these technical difficulties, some new imaging technologies have been developed to improve spatial resolution, such as the Imaging Cryomicrotome (Krueger et al., 2013), micro-PET/CT hybrid systems (Gargiulo et al., 2012), and magnetic resonance (MR) tagging (Epstein et al., 2002; Thomas et al., 2004). Researchers must consider these fundamental differences in anatomy and cardiac kinetics across species when interpreting the animal study results as they give rise to different phenotypes between humans with genetic predispositions and transgenic animal models that recapitulate the diseases (Riehle and Bauersachs, 2019). Consideration of available options for post-operative evaluation must be made when choosing an animal model as well. Large animals, such as swine and sheep, which are anatomically and physiologically closer to the humans, are used to minimize these phenotypic differences between humans and animal models. In MI research, it is essential to correctly identify the perfusion and coronary collateral circulation systems in the animal of choice, as the variations in these structures across animals can significantly affect the early and progressive response to ischemia (Harken et al., 1981; Hill and Iaizzo, 2009). In this regard, swine and ovine models are preferred to smaller animals, such as rodents and canines, as their coronary arterial structure and scant collateral arteries resemble those of humans, which allows for the creation of predictable infarct size at a preferred location in the myocardium (Dixon and Spinale, 2009; Nguyen and Wu, 2015). Moreover, swine, sheep, and human myocardia share high degrees of similarities in cardiac kinetics (Milani-Nejad and Janssen, 2014) and healing characteristics following injury (Lelovas et al., 2014). A domestic sheep is ideal in size for clinical imaging modalities (such as MRI and CT) and medical devices (such as pacemakers and stents) designed for the humans (Ribitsch et al., 2020).

However, there are several disadvantages of using large animal models, which can eventually limit the reproducibility of the research. Some of the factors that discourage their use in research are the high cost required for performing the experiments, housing/maintenance and care, and lower acceptance as model animals by society (Freedman et al., 2015; Camacho et al., 2016; Spannbauer et al., 2019). The public’s growing concern about the welfare of research animals, especially companion animals such as dogs and cats, has led to more stringent laws, policies, and guidelines, limiting their prevalent use in research (National Research Council (Us) Committee on Scientific and Humane Issues in the Use of Random Source Dogs and Cats in Research, 2009). Additionally, swine, especially the Yorkshire pigs, dramatically gain weight in adulthood, which complicates long-term follow-up and makes it an unsuitable model for chronic IHF studies (Schuleri et al., 2008; Tohyama and Kobayashi, 2019). Anesthetized swine of MI models often display high mortality rates due to fatal arrhythmia, such as ventricular fibrillation, during or shortly after the coronary artery occlusion or ischemia (Halkos et al., 2008; Lim et al., 2018), which may introduce sample size bias and confound experimental results. Table 1 shows a comparative analysis of different animal models commonly used in MI studies.

**TABLE 1:** Comparison of central cardiovascular systems in small and large animals used in MI study.

Animal	*Body weight (kg)	*HR (rpm) *BP (mmHg)	**Coronary anatomy	**Collaterals	***Advantages/Similarities to human	***Disadvantages/Dissimilarities to human
Mouse/Rat (Rodents)	**Mouse:** 0.02–0.063 **Rat:** 0.225–0.52	**Mouse:** • HR: 310–840 • SBP:113–160 • DBP: 81–11 **Rat:** • RHR: 250–493 • SBP: 84–184 • DBP: 58–145	• Distinct septal coronary artery coursing along the right interventricular septum and a left coronary artery → Result in different regionality of infarction compared with human and large animals	• Have collateral arteries → Vessel occlusion does not cause a complete cessation of circulation • Mice – Collateral extent varies widely within the species primarily due to variation at a single genetic locus	• Transgenic models readily available (e.g., atherosclerosis model) • Express proteins with similar functions and roles as those in humans • Lower cost for maintenance • Similar electrophysiological characteristics and calcium transport	• Most remote from human contractile function due to small size and short lifespan • Visualization and histological assessment are difficult due to the small coronary arteries • Hearts function at very high HRs • Ventricular CMs predominately express fast α-MHC (>94–100%)
Rabbit	1–6	• HR: 130–300 • SBP: 90–130 • DBP: 60–90	• Left dominance • The LCx is larger and supplies a much greater portion of the myocardium than does LAD	• Have little innate coronary collateral flow	• Less expensive than other large animal models • Transgenic models available • Similar electrophysiological characteristics and calcium transport	• Their kinetics of cardiac contraction and relaxation are still very faster than those of humans • Different and inconsistent coronary artery systems • Not always considered as large animal • Less reported studies than other species • No tricuspid valve
Dog (Canine)	7–16	• HR: 70–160 • SBP: 95–136 • DBP: 43–66	• Left dominance	• Variable and extensive preexisting collateral epicardial circulation which can supply as much as 40% of the blood flow after the occlusion of a coronary artery	• Similar electrophysiological characteristics and calcium transport • Similar excitation-contraction coupling processes • Similar ventricular activation sequence	• Difficult to obtain the necessary approval for using canines as an animal model • Extensive collateral circulation in myocardium → Cannot create consistent degrees of MI → Different ischemic patterns than other large mammalians → Delivers blood flow preferentially to the epicardial tissue, thus at the greater vulnerability of the endocardium to necrosis and the phenomenon of the “wave front of cell death”
Sheep (ovine)	20–160	• HR: 60–120 • SBP: ∼90–115 • DBP: ∼100	• Left dominance	• Have little innate coronary collateral flow	• Scant collateral arteries, allowing to produce a predictable infarct size	• Costly experiment and maintenance • High risk of arrhythmia, including fibrillation, with little provocation • Dissimilar coronary anatomy • Difficult to perform non-invasive due to thoracic and gastrointestinal anatomy • High risk of arrhythmia, including fibrillation • High risk of infection
Pig (swine/Porcine)	200–300	• HR: 50–116 • SBP: 135–150 • DBP: –	• Right dominance • Like human, left coronary artery larger in diameter, and longer than the right coronary artery	• Scant innate collateral arteries, primarily localized to the mid myocardium and subendocardium (little collateral blood flow)	• Myocardial excitation-contraction coupling • *In vivo* contractile and relaxation kinetics • Similar coronary anatomy and gross anatomical structure to humans • Similar cardiac output to humans • Scant collateral arteries, allowing to produce a predictable infarct size • Resistant to infections and relatively rapid healing after surgery	• Costly experiment and maintenance • High risk of arrhythmia, including fibrillation, with little provocation • Different ventricular activation sequence is different due to different distribution of Purkinje fibers • Heart-to-body ratio decreases with aging → Gain weight dramatically in their adulthood, thus not suitable for long-term study • Brief diastole makes them prone to coronary insufficiency and increase sensitivity and decrease specificity the effects of drugs or treatment
Miniature Pig (mini swine)	32–68	• HR: ∼ 56 • SBP: 122 ± 16 • DBP: 88 ± 10	• Right dominance • The posterior descending artery arise from right coronary artery	• Have little coronary collateral flow	• Similar heart-to-body weight ratio • Similar coronary artery distribution • Cardiac anatomy, metabolism, electrophysiology – comparable to man • Relatively smaller body size than large pig, even at full sexual maturity → Offer experimental control and reproducibility due to manageable size	• Similar to large pig (above)
Human	50–86	• HR: 60–100 • SBP: 115–135 • DBP: 60–80	• Right dominance • Left coronary artery larger in diameter and longer than the right coronary artery	• Minimal preexisting collaterals		

*Cardiovascular parameters (Body weight, HR, and BP) are retrieved from Stubhan et al. (2008); Bode et al. (2010), Gandolfi et al. (2011); Milani-Nejad and Janssen (2014). **The characteristics of coronary and collateral artery anatomy are adapted from Blair (1961); Spadaro et al. (1980), Weaver et al. (1986); Maxwell et al. (1987), Kamimura et al. (1996); Podesser et al. (1997), Hearse (2000); Kumar et al. (2005), Dixon and Spinale (2009); Lelovas et al. (2014). ***Other characteristics are adapted from Harken et al. (1981); Khan (1984), Hearse (2000); Nunoya et al. (2007), Dixon and Spinale (2009); Milani-Nejad and Janssen (2014), Morrissey et al. (2017); Stricker-Krongrad et al. (2017), Tang et al. (2018). SBP, systolic blood pressure; DBP, diastolic blood pressure; LCx, left circumflex coronary artery; LAD, left anterior descending coronary artery; CM, cardiomyocyte.

No single animal model can sufficiently answer every question raised in the field of cardiovascular research. Different species as animal models for MI studies may vary in size, anatomical structure, and genetic and phenotypic expression, and have their own advantages and disadvantages. Because of the heterogeneity and multimorbidity observed in patients with MI, animal models in the preclinical studies are considered by some as too remote to be applicable in translational efforts. Some researchers emphasize the use of human-based research methods, such as the use of human-induced pluripotent stem cells (iPSCs), cardiac organoids, and cardiovascular “organs-on-chips” (Ribas et al., 2016; Pound and Ritskes-Hoitinga, 2018; Richards et al., 2020). However, it is undeniable that there is no adequate substitute for animal models that allow us to systematically examine how the entire body systems respond to a disease. The ideal approach to preclinical studies would be to use multiple, complementary animal models, and human-based models to utilize the advantages of strengths of each model and take preventive measures to minimize bias in the experimental design and data interpretation.

### Seeing What We Want to See: Biased Experiments in Animal Studies Decelerate Reliable Clinical Translations in MI Studies

A rapid technological advancement has dramatically improved our understanding of human heart diseases and therapeutic development; however, the translation of these findings has not been keeping up with this trend (Ioannidis, 2005, 2016). This review argues that two primary sources of this slow translation are: (1) the lack of transparency in experimental design and data assessment and (2) excessive variation in the protocols for animal surgeries; both of these factors may have resulted from the inherent technical difficulties in dealing with large animal models. Compared to small animals, a higher degree of financial, husbandry, and technical obstacles exist in large animal studies, which often limit the study scale and lead to self-justified modifications in the surgical protocols along with lack of internal validity. Studies involving large animal models are expensive and technically demanding as they require advanced surgical and anesthetic techniques and materials. However, most published papers do not report the precise and detailed protocols or visual representations needed for other researchers to reproduce the same animal model or verify the surgical procedure and experimental results. The difficulty in finding a verifiable open reference leads to poor experimental designs and varied animal survival rates; this introduces sampling bias, which is especially detrimental for small-scale studies involving large animal models.

**FIGURE 1:**
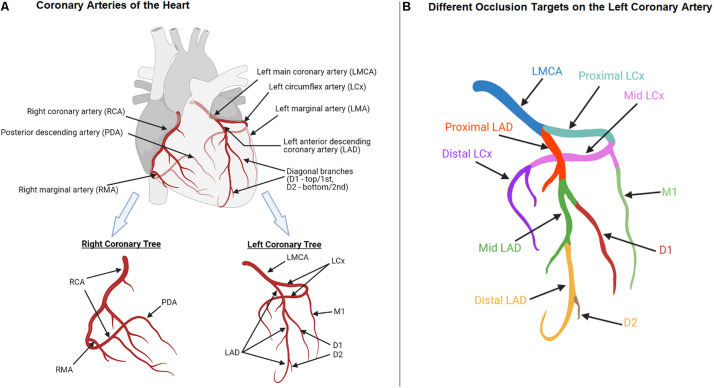
Anatomy of coronary arteries of the heart **(A)** Left and right coronary trees; **(B)** Different occlusion targets (indicated in different colors) on the left coronary artery.

Another source of bias is the flexibility in surgical procedures for creating MI in animals. For example, the most common method to induce acute MI is the permanent or catheter-assisted temporary coronary artery occlusion with the left anterior descending coronary artery (LAD) as the primary target vasculature. The mortality rate from LAD occlusion is relatively high, especially for large animals, as they are at a considerable risk of developing ventricular fibrillation following MI (de Jong et al., 2014; Mu et al., 2016; Lim et al., 2018). To avoid this occurrence, the left circumflex artery (LCx) is often used as an alternative target at the cost of inducing a smaller infarct at a different location (Hirano et al., 2017; Cremer et al., 2019). The substantial inconsistency in the occlusion site along these two coronary arteries further complicates the MI studies. Some segments of the LAD and LCx commonly targeted for occlusion are as follows and can be found in Figure 1:

• The LAD “distal to” the 1st diagonal branch (Kraitchman et al., 2003; Okura et al., 2012; Li et al., 2013; Sharp et al., 2017).
• The “Mid”-LAD “just beyond” the 1st diagonal branch (Lim et al., 2018).
• The “Mid-left” LAD “distal to” the 1st diagonal branch (de Jong et al., 2014).
• The LAD “beyond” the 1st diagonal branch (Wolf et al., 2009).
• The LAD “distal to” the 2nd diagonal branch (Rabbani et al., 2008; Wang et al., 2009; Mu et al., 2016; Rabbani et al., 2017).
• The “proximal” LCx (Timmers et al., 2011; Wang B. et al., 2017).
• The 1st “marginal” branch of the LCx (Gálvez-Montón et al., 2014).

Several other studies have not specified the exact location of occluded segments of the LAD (Wolf et al., 2009; Crisostomo et al., 2019) or LCx (van der Velden et al., 2004; Charles et al., 2020). Without appropriate visual representation, this inconsistent and vague language, such as “beyond” and “mid,” leaves room for arbitrary interpretation and changes in the surgical procedures, potentially leading to varied experimental outcomes. One study determined the site(s) and number of ligatures based on the visual inspection of the LAD and LCx branches in each ovine, in order to produce a consistent anterolateral infarct size across different animals (Locatelli et al., 2011) while many studies have not reported the infarct size (Kraitchman et al., 2003; Zhao et al., 2014; Alestalo et al., 2015; Haller et al., 2015; Kim et al., 2017; Lim et al., 2018; Ishida et al., 2019).

Additionally, different surgical procedures for inducing MI often create various degrees of ischemia via different pathogenic mechanisms thus generating different MI models (Table 2). For example, catheter-based occlusion is often used as a non-invasive way to induce MI, but there is a significant variation in the occlusion sites and durations followed by reperfusion across different studies (Table 3). Some studies using pig models have demonstrated that the longer occlusion duration resulted in bigger infarct sizes and more severe left ventricular dysfunction (Garcia-Dorado et al., 1987; Ghugre et al., 2013; Thomas et al., 2021). However, besides the occlusion site and duration, this inconsistent infarct size and ventricular remodeling were likely to be affected by the subsequent reperfusion. Myocardial reperfusion using thrombolytic therapy or primary percutaneous coronary intervention is a treatment option for human MI patients. However, it is known that the reperfusion of myocytes irreversibly injured by ischemia following coronary occlusion may accelerate the necrotic process, a phenomenon called “myocardial ischemia-reperfusion injury.” This could consequently affect the infarct size and lead to adverse cardiac remodeling (Braunwald and Kloner, 1985; Yellon and Hausenloy, 2007; Hausenloy and Yellon, 2013; Acharya, 2020). All these situational specifics of a surgical procedure as part of MI preclinical study design (for example, method, site, and duration of coronary artery occlusion, and presence and duration of reperfusion following occlusion) potentially limit the generalizability and reproducibility of scientific results and likely contribute to the failure of subsequent clinical trials.

**TABLE 2:** Comparison of surgical procedures used to induce MI in large animal models.

MI induction methods	Open-chest (or surgical-based) vs. Close-chest (or catheter based)	Advantages	Disadvantages	Studies
Coronary artery ligation	Open-chest	• Provides precise timing, location and extent of the coronary event due to direct visualization and observation of the procedure and targeted area of infarct	• Invasive procedure – Increased mortality and complications – Affects the whole balance of bodily function and modifies local and systemic immunological and inflammatory responses • The site of ligation of the vessel varies (proximal, mid, or distal) in studies, resulting in various degree of ischemic injury and mortality rate • The LAD occlusion often causes ventricular fibrillation and sudden death, especially in pigs (Muller et al., 1988)	LAD ligation (Krause et al., 2007; Wolf et al., 2009; Chen et al., 2014; de Jong et al., 2014; Zhao et al., 2014; Haller et al., 2015; Lim et al., 2018) LCx ligation (van der Velden et al., 2004; Timmers et al., 2011; Gálvez-Montón et al., 2014; Wang L. et al., 2017; Charles et al., 2020)
Ameroid constrictor or hydraulic occluder	Open-chest or close-chest	• Gradual occlusion mimics chronic MI and enables the development of the collateral arterial supply	• May require invasive procedure	Sjaastad et al., 2000; Shudo et al., 2011; Potz et al., 2018; Ishida et al., 2019
Cryoinjury	Open-chest	• Freezing-induced scar has similar cellular patterns of coagulation necrosis of MI – a suitable model used to demonstrate myocardial repair, heart regeneration and cellular remodeling using cellular therapies	• Invasive procedure • The pathophysiology of freezing induced MI is different from other methods because acute cell death occurs following the cryoinjury without concomitant ischemia • Several applications are necessary for large animal hearts and also due to rapid defrosting of cryoprobe, which makes it difficult to control the size of infarction • Difficult to induce transmural infarction • Less tested in large animals	Yang et al., 2010, 2012; Hirano et al., 2017
Percutaneous intracoronary embolization using various insertion materials, followed by reperfusion	Close-chest	• Minimally invasive • Resembles human course of atherosclerotic disease superimposed by thrombus formation during MI event • Various embolic agents – Sponge foam/sponge microspheres, coils, polystyrene microspheres, alcohol injection, balloon catheter • Clinically relevant as myocardial reperfusion is performed in human MI by fibrinolytic therapy or Percutaneous Coronary Intervention (PCI) • Timely reperfusion of the coronary artery after MI helps salvage the viable myocardium, limit infarct size, preserve LV systolic function and prevent the onset of heart failure	• Requires anticoagulant therapy to prevent blood clot formation during instrumentation • Require anti-arrhythmic protocol to prevent arrhythmia and ventricular fibrillation • Difficult to control the exact location, length and duration of the coronary artery occlusion and the overall volume of myocardial necrosis (Camacho et al., 2016) • Requires advanced technical skills and highly trained personnel to manipulate the catheter for deployment of the material for embolization • Inconsistent occlusion duration across studies • Reperfusion of an ischemic area often results in myocardial cell necrosis (or called reperfusion injury) • Just like coronary artery ligation, mortality rate can vary depending on the embolization sites (higher mortality at the proximal site)	Sponge foam/sponge microspheres (Dariolli et al., 2014; Sun et al., 2020) Coils (Watanabe et al., 1998; Li et al., 2000; Makkar et al., 2005; Dib et al., 2006) Polystyrene microspheres (Hanes et al., 2015; Suzuki et al., 2016) Ethyl alcohol injection (Joudinaud et al., 2005; Rienzo et al., 2020) Balloon catheter (Kraitchman et al., 2003; Price et al., 2006; Wang et al., 2009; Lu et al., 2012; Okura et al., 2012; Li et al., 2013; de Jong et al., 2014; Mu et al., 2016)
Chemical reagent e.g., isoproterenol	Closed-chest Subcutaneously intraperitoneally, intravenously	• Non-invasive – can be injected subcutaneously intraperitoneally, or intravenously • Low mortality rate	• Indirect method – unable to visualize the effects on the targeted area during the procedure • Different methods of administration affect the drug metabolism and its conversion into inactive metabolites • Less tested in large animals	Kim et al., 2014; Lim et al., 2014

*Comparison is adapted from Halim et al. (2018).*

**TABLE 3:** Variations in occlusion duration in catheter-based MI studies.

Large animal model	MI induction method	Occlusion location	Occlusion duration	Mortality rate due to MI occlusion (during or shortly after MI induction)	Infarct size (Untreated group)	Study
Farm pigs	Cardiac catheterization (carotid sheath and coronary angioplasty balloon)	LAD beyond the first diagonal branch	60 min	–	–	Kraitchman et al., 2003
Young Yorkshire pigs	Percutaneous transluminal angiography (balloon occlusion) followed by reperfusion (after anticoagulation)	LAD distal to the second diagonal branch	60 min	Four died within first 60 min after coronary occlusion due to ventricular fibrillation	8.8 ± 2.1%	Wang et al., 2009
Farm pigs	Balloon occlusion/reperfusion	LAD just distal to the second largest diagonal branch	60 min	Six died within 3 days after MI	5.3 ± 1.8%	Price et al., 2006
Yorkshire-cross bred pigs	Balloon catheter occlusion-reperfusion	LAD just distal to the second diagonal branch	60 min	Two died within the first 15 min of reperfusion due to ventricular arrhythmias	9.8 ± 1.1	Techiryan et al., 2018
Yorkshire pigs	Angioplasty-induced coronary artery occlusion-reperfusion	Proximal LAD at the level of the first or second diagonal branch	75 min	17% developed fatal arrhythmias during ischemia	8.1 ± 1.8% in Control	Halkos et al., 2008
Chinese mini-pigs	Acute MI – percutaneous transluminal angiography (balloon occlusion) followed by reperfusion (after anticoagulation)	LAD distal to the second diagonal	90 min	Four died due to ventricular fibrillation during occlusion procedure	56% decreased after ILK-MSC treatment (*P* < 0.001) (<40% decrease after treatment of MSC alone)	Mu et al., 2016
Landrace pigs	Moderate acute MI by inflation of an angioplasty balloon	Left circumflex artery occlusion (posterolateral infarct)	90 min	Two died of ventricular fibrillation (VF) 1 day post-MI	9.6 ± 1.3%	de Jong et al., 2014
Yorkshire pigs	Percutaneous balloon dilation catheter	LAD distal to second diagonal branch	• 45 min • 90 min	–	– Result: More adverse remodeling in the 90-min groups than 45-min groups	Ghugre et al., 2013
Yorkshire pigs	Occlusion-reperfusion	Mid LAD	• 60 min • 90 min	–	–	Thomas et al., 2021
Large white pigs	Occlusion-reperfusion	Mid LAD	• 30 min • 45 min • 60 min • 90 min • Permanent	One died from ventricular fibrillation during coronary occlusion One from 30 min group and one from 60 min group died the night after the occlusion One developed malignant hyperthermia	LV mass: • 30 min: 0.46 (0.42)% • 45 min: 2.85 (1.14)% • 60 min: 9.74 (1.65)% • 90 min: 8.93 (1.37)% • Permanent: 3.17 (1.17)% Transmural extension: • 30 min: 14.6 (11.4)% • 45 min: 42.1 (12.9)% • 60 min: 87.4 (6.6)% • 90 min: 96.2 (3.2)% • Permanent: 100 (0)% Result: Infarct size and the transmurality index correlated exponentially with the duration of the occlusion	Garcia-Dorado et al., 1987
Ovine (Sheep)	Coronary artery ischemia–reperfusion Coronary occlusion using a suture loop	Mid-second diagonal branch	90 min	One died of ventricular fibrillation during the ischemic period	–	Dayan et al., 2016
Coopworth ewes (Sheeps)	PTCA Balloon occlusion-reperfusion vs. Thrombogenic coil embolization (permanent)	Distal to the first diagonal branch but proximal to the second diagonal branch	90 min (coil: 2 min)	Two died of arrhythmia within 4 min following reperfusion Coil embolization group: Three died 30 min, 60 min, and between 6 and 12 h post-infarction	– Result: Restriction of coronary artery occlusion to 90 min results in infarction, but less LV dysfunction with reduced early remodeling, compared with permanent occlusion	Charles et al., 2000
Sheep	Balloon occlusion-reperfusion	Mid-LAD	90 min	Phase 3: 34/68 sheep died during infarct induction due to ventricular fibrillation refractory to defibrillation	18.4 ± 1.5%	Houtgraaf et al., 2013

### Potential of Human-Based Models as an Alternative for Animal Models?

Whether small or large, animal models cannot fully recapitulate human CVD phenotypes, thus requiring new forms of human-based experimentation. The tissue engineering community has been developing *in vitro* and *in silico* CVD models for more physiologically and clinically relevant readouts of CVDs (Savoji et al., 2019). Human organs-on-chips are 3D microfluidic cell culture devices that mimic the physical and mechanical microenvironment of key organ systems and provide dynamic vascular perfusion *in vitro*, which is difficult to achieve in 2D cell culture (Ingber, 2018). This burgeoning biomimetic system can incorporate patient-specific cell models, allowing the study of pathophysiology and pharmacological responses unique to each patient (Ingber, 2020; Wu et al., 2020).

However, an organ-on-a-chip is still limited in that although it can capture distinct functional units of organ systems separately (e.g., heart vs. liver), it cannot link each unit via vascular channels (e.g., the hepatic portal system). “Multi” organ-on-a-chip device may allow combining several cellular models in a single chip; however, certain technical difficulties, such as selecting a co-culture medium required for incorporating multiple cell lineages and ensuring the correct sizing of each organ, need to be resolved (Bovard and Sandoz, 2020). However, this innovative *in vitro* model is still distant from a complete replacement of animal studies because they cannot mimic the complex nervous and immune systems of humans. Thus, investigators, particularly those concerned with cognition, behavior, immune responses, and pain management, still require animal studies to systematically monitor disease progression and develop corresponding therapeutic interventions. Animal studies have been misinterpreted as poor predictors of clinical study outcomes. This may be true merely because animals and humans are inherently different, and the human body and pathogenesis of CVDs and other diseases are far too complicated to be replicated in other models. However, this inherent difficulty should not be used as an excuse to adopt a less rigorous but more convenient experimental design and data interpretation. Although new technological advances will allow us to adopt more disease-representative models, the clinical study outcomes will still largely depend on scientific rigor.

## Discussion

Despite increasing knowledge about the etiologies of MI and relevant therapeutic strategies, the translational gap between basic science and clinical research is widening. Lack of experimental rigor and quality in preclinical research has been accused as the main cause of slow translation of “promising” preclinical results, and various issues regarding reproducibility have been raised across different biomedical and social science fields (Pound et al., 2004; Begley and Ioannidis, 2015).

In section “Suitability of Animal Models for Human MI,” we discussed the importance of choosing a representative animal model in preclinical studies and considering the differences between different animal species and humans when interpreting experimental data. Some researchers believe that the limited opportunities to carry out studies based on large animal models prevent them from testing their hypothesis more rigorously and openly, justifying adjustments in an experimental design and biased interpretations of study outcomes. Yet, the discordance between animal-based preclinical and human-based clinical studies is often attributed for the failures of clinical trials for cardiovascular and other disease therapies (Pound et al., 2004; Perel et al., 2007). Some human-based preclinical models have been proposed as a complementary platform to overcome the limitations of using an animal model. However, they will not replace animal models entirely soon as discussed in section “Potential of Human-Based Models as an Alternative for Animal Models?” The difficulty of establishing the optimal animal model prompts a periodic systematic review or meta-analysis of animal studies (Sandercock and Roberts, 2002; Pound et al., 2004; Hooijmans and Ritskes-Hoitinga, 2013). However, a systematic review of studies with poor methodological quality is likely to produce additional animal studies of similarly poor quality. Instead, the preclinical, animal study quality must be scrutinized at the original study design process and journals’ review process at the time of submission.

In section “Seeing What We Want to See: Biased Experiments in Animal Studies Decelerate Reliable Clinical Translations in MI Studies,” we reviewed how the lack of standardized protocols and transparency in preclinical MI studies involving animal experiments could allow investigators too much flexibility in their study design and data assessment, depriving “promising” preclinical research results reproducibility and translational power. Investigators often adopt a disease model that is remote from what they intend to model and tend to report the desired results that are harmonious with their hypothesis alone (Baker, 2016). A standardized experimental method is critical for ensuring reproducibility, but the lack of overall methodological rigor in preclinical cardiovascular studies is prevalent, delaying the translational process; this issue has called for a set of improved reporting standards, more strict funding policies, and better instructions for peer reviewer (Hooijmans et al., 2010; Hirst and Altman, 2012; Henderson et al., 2013; Anon, 2013; Principles and Guidelines for Reporting Preclinical Research and National Institutes of Health (NIH), 2021).

Four elements of methodological quality of preclinical research that critically determine its translational power are randomization, sample size calculation, blinding, and heterogeneity of animals used (i.e., strains, ages, and sexes) (Henderson et al., 2013). A recently added critical element of heterogeneity of animal models is environmental factors, which suggests the benefit of multi-laboratory experiments (Richter et al., 2009; Voelkl et al., 2018). Ramirez et al. (2017) found that randomization was reported only in 21.8%, blinding in 32.7%, and sample size estimation in 2.3% of all preclinical cardiovascular studies published in five leading cardiovascular journals between July 2006 and June 2016 (Ramirez et al., 2017). Similar or worse results are found in the review of thirty-one systematic reviews of animal studies on treatments for various diseases (Hirst et al., 2014; van Luijk et al., 2014). Additionally, the quality of these study design elements has not improved in all disease-specific studies, except for stroke research (Hirst et al., 2014). From 1997 to 2007, the number of cardiovascular papers and journals increased by 56.9 and 75.2%, respectively, yet 46% of original papers published in cardiovascular journals in the same period were poorly cited (with < = 5 citations in the 5 years following publication); however, 44% of cardiovascular journals had more than three-fourths of the journal’s content poorly cited at 5 years (Ranasinghe et al., 2015). Interestingly, studies that employed randomization, blinding, or sample size estimation were equally cited in numbers as those that did not; however, studies that included both males and females were less frequently cited, suggesting that methodological rigor might have been overlooked by cardiovascular researchers (Ramirez et al., 2017). This suggests the need for strict enforcement of a comprehensive guideline and requirements by journals and funding institutions to ensure the rigor of animal studies and publication to the level of human-involving, clinical studies, which consequently promotes reproducibility and animal welfare (Hooijmans et al., 2010; Carbone and Austin, 2016).

It is almost always impossible to control every aspect of a scientific experiment and to perfectly mimic human pathophysiology in a disease model. Consequently, any experimental data are biased, and it is a matter of how biased they are and whether researchers are aware of and report those biases correctly. Additionally, the failure to reproduce or conflicting data is not always a vice but could be a valuable resource that potentially enriches biomedical research (Daugherty et al., 2016). However, in translational medicine, reproducibility is the ultimate goal, and this review article emphasizes there is much room for improvements in preclinical study design and animal models for MI research. Methodological rigors such as sample randomization, consistent surgical procedures, blind analyses, and greater sample statistical power are essential in animal models of human CVDs or other diseases. Along with following the correct procedures during research, transparent reporting of experimental protocols and results is equally essential to improve reproducibility, effectiveness, predictability, and safety of the clinical studies.

Considering the economic and emotional cost of a clinical trial and the exponentially growing number of published articles, it may be much more cost-effective from the standpoint of the entire population to maintain rigor and quality in the preclinical study level with good practice and additional cost than to see a series of “promising” preclinical study continuously failing in clinical trials (Freedman et al., 2015). However, probably most trained researchers may be well aware of these prerequisites of successful translation mentioned above. The root cause of the imbalance between the translational crisis and exponentially growing research in the cardiovascular field might be the competition for grants and positions (Baker, 2016). In this case, more opportunities for quality training and mentorship within research communities as well as a clear publication or funding guideline by journals and funding institutions are proposed (Begley and Ioannidis, 2015).

Yet, probably most trained researchers may be well aware of these prerequisites of successful translation. The root cause of the imbalance between the translational crisis and exponentially growing research in cardiovascular field might be the competition for grants and positions (Baker, 2016). In this case, more opportunities for quality training and mentorship within research communities in addition to a clear publication or funding guideline by journals and funding institutions are proposed (Begley and Ioannidis, 2015).

## Author Contributions

HSy, HSe, and YS: conceptualization, writing—review and editing, and visualization. HSy and HSe: methodology, investigation, and writing—original draft preparation. HSy and YS: resources and project administration. YS: supervision and funding acquisition. All authors have read and agreed to the published version of the manuscript.

## Conflict of Interest

The authors declare that the research was conducted in the absence of any commercial or financial relationships that could be construed as a potential conflict of interest.

## References

[B1] Acharya D. (2020). Unloading and reperfusion in myocardial infarction. *Circulation* 13:1. 10.1161/CIRCHEARTFAILURE.119.006718 31959017

[B2] Alestalo K. Korpi R. Mäkelä J. Lehtonen S. Mäkelä T. Yannopoulos F. (2015). High number of transplanted stem cells improves myocardial recovery after AMI in a porcine model. *Scand. Cardiovasc. J.* 49 82–94. 10.3109/14017431.2015.1018311 25705991

[B3] Baker M. (2016). 1,500 scientists lift the lid on reproducibility. *Nature* 533 452–454. 10.1038/533452a 27225100

[B4] Begley C. G. Ioannidis J. P. A. (2015). Reproducibility in science: improving the standard for basic and preclinical research. *Circ. Res.* 116 116–126. 10.1161/circresaha.114.303819 25552691

[B5] Blair E. (1961). Anatomy of the ventricular coronary arteries in the dog. *Circ. Res.* 9 333–341. 10.1161/01.res.9.2.333

[B6] Bode G. Clausing P. Gervais F. Loegsted J. Luft J. Nogues V. (2010). The utility of the minipig as an animal model in regulatory toxicology. *J. Pharmacol. Toxicol. Methods* 62 196–220. 10.1016/j.vascn.2010.05.009 20685310

[B7] Bolli R. Becker L. Gross G. Mentzer R. Balshaw D. Lathrop D. A. (2004). Myocardial protection at a crossroads: the need for translation into clinical therapy. *Circ. Res.* 95 125–134. 10.1161/01.res.0000137171.97172.d7 15271864

[B8] Bovard D. Sandoz A. (2020). *How to build your multiorgan-on-a-chip system: a case study. Organ-on-a-chip.* Amsterdam: Elsevier, 463–506.

[B9] Bracken M. B. (2009). Why animal studies are often poor predictors of human reactions to exposure. *J. R. Soc. Med.* 102 120–122. 10.1258/jrsm.2008.08k033 19297654 PMC2746847

[B10] Braunwald E. Kloner R. A. (1985). Myocardial reperfusion: a double-edged sword? *J. Clin. Invest.* 76 1713–1719. 10.1172/jci112160 4056048 PMC424191

[B11] Camacho P. Fan H. Liu Z. He J.-Q. (2016). Large mammalian animal models of heart disease. *J. Cardiovasc. Dev. Dis.* 3:4. 10.3390/jcdd3040030 PMC5715721 29367573

[B12] Cannon R. O. (2005). Mechanisms, management and future directions for reperfusion injury after acute myocardial infarction. *Nat. Clin. Pract. Cardiovasc. Med.* 2 88–94. 10.1038/ncpcardio0096 16265379

[B13] Carbone L. Austin J. (2016). Pain and laboratory animals: publication practices for better data reproducibility and better animal welfare. *PLoS One* 11:e0155001. 10.1371/journal.pone.0155001 27171143 PMC4865140

[B14] Charles C. J. Elliott J. M. Nicholls M. G. Rademaker M. T. Richards M. (2000). Myocardial infarction with and without reperfusion in sheep: early cardiac and neurohumoral changes. *Clin. Sci.* 98 703–711. 10.1042/cs19990266 10814608

[B15] Charles C. J. Li R. R. Yeung T. Mazlan S. M. I. Lai R. C. de Kleijn D. P. V. (2020). systemic mesenchymal stem cell-derived exosomes reduce myocardial infarct size: characterization with mri in a porcine model. *Front. Cardiovasc. Med.* 7:601990. 10.3389/fcvm.2020.601990 33304934 PMC7701257

[B16] Chen C.-H. Chang M.-Y. Wang S.-S. Hsieh P. C. H. (2014). Injection of autologous bone marrow cells in hyaluronan hydrogel improves cardiac performance after infarction in pigs. *Am. J. Physiol. Heart Circ. Physiol.* 306 H1078–H1086. 24508641 10.1152/ajpheart.00801.2013

[B17] Chong J. J. H. Yang X. Don C. W. Minami E. Liu Y.-W. Weyers J. J. (2014). Human embryonic-stem-cell-derived cardiomyocytes regenerate non-human primate hearts. *Nature* 510 273–277. 24776797 10.1038/nature13233 PMC4154594

[B18] Christman K. L. Lee R. J. (2006). Biomaterials for the treatment of myocardial infarction. *J. Am. Coll Cardiol.* 48 907–913. 10.1016/j.jacc.2006.06.005 16949479

[B19] Cremer S. Schloss M. J. Vinegoni C. Zhang S. Rohde D. Feruglio P. F. (2019). A mouse model of recurrent myocardial infarction reports diminished emergency hematopoiesis and cardiac inflammation. *BioRxiv* 2019:4. 10.1016/j.jacc.2019.12.056 PMC7254576 32130926

[B20] Crisostomo V. Baez C. Abad J. L. Sanchez B. Alvarez V. Rosado R. (2019). Dose-dependent improvement of cardiac function in a swine model of acute myocardial infarction after intracoronary administration of allogeneic heart-derived cells. *Stem Cell Res. Ther.* 10:152. 31151405 10.1186/s13287-019-1237-6 PMC6544975

[B21] Dariolli R. Takimura C. K. Campos C. A. Lemos P. A. Krieger J. E. (2014). Development of a closed-artery catheter-based myocardial infarction in pigs using sponge and lidocaine hydrochloride infusion to prevent irreversible ventricular fibrillation. *Physiol. Rep.* 2:8. 10.14814/phy2.12121 PMC4246577 25168871

[B22] Daugherty A. Hegele R. A. Mackman N. Rader D. J. Schmidt A. M. Weber C. (2016). Complying with the national institutes of health guidelines and principles for rigor and reproducibility: refutations. *Arterioscler. Thromb. Vasc. Biol.* 36 1303–1304. 10.1161/atvbaha.116.307906 27335467

[B23] Dayan V. Sotelo V. Delfina V. Delgado N. Rodriguez C. Suanes C. (2016). Human mesenchymal stromal cells improve cardiac perfusion in an ovine immunocompetent animal model. *J. Invest. Surg.* 29 218–225. 10.3109/08941939.2015.1128997 26891475

[B24] de Jong R. van Hout G. P. J. Houtgraaf J. H. Kazemi K. Wallrapp C. Lewis A. (2014). Intracoronary infusion of encapsulated glucagon-like peptide-1-eluting mesenchymal stem cells preserves left ventricular function in a porcine model of acute myocardial infarction. *Circ. Cardiovasc. Interv.* 7 673–683. 10.1161/circinterventions.114.001580 25294400

[B25] Dib N. Diethrich E. B. Campbell A. Gahremanpour A. McGarry M. Opie S. R. A. (2006). percutaneous swine model of myocardial infarction. *J. Pharmacol. Toxicol. Methods* 53 256–263. 16460969 10.1016/j.vascn.2005.10.005

[B26] Dirksen M. T. Laarman G. J. Simoons M. L. Duncker D. J. G. M. (2007). Reperfusion injury in humans: a review of clinical trials on reperfusion injury inhibitory strategies. *Cardiovasc. Res.* 74 343–355. 10.1016/j.cardiores.2007.01.014 17306241

[B27] Dixon J. A. Spinale F. G. (2009). Large animal models of heart failure: a critical link in the translation of basic science to clinical practice. *Circ. Heart Fail.* 2 262–271. 10.1161/circheartfailure.108.814459 19808348 PMC2762217

[B28] Downey J. M. Cohen M. V. (2009). Why do we still not have cardioprotective drugs? *Circ. J.* 73 1171–1177. 10.1253/circj.cj-09-0338 19506318

[B29] Epstein F. H. Yang Z. Gilson W. D. Berr S. S. Kramer C. M. French B. A. M. R. (2002). tagging early after myocardial infarction in mice demonstrates contractile dysfunction in adjacent and remote regions. *Magn. Reson. Med.* 48 399–403. 10.1002/mrm.10210 12210951

[B30] Ferreira G. S. Veening-Griffioen D. H. Boon W. P. C. Moors E. H. M. van Meer P. J. K. (2020). Levelling the translational gap for animal to human efficacy data. *Animals* 10:7. 10.3390/ani10071199 PMC7401509 32679706

[B31] Freedman L. P. Cockburn I. M. Simcoe T. S. (2015). The economics of reproducibility in preclinical research. *PLoS Biol.* 13:e1002165. 10.1371/journal.pbio.1002626 26057340 PMC4461318

[B32] Gálvez-Montón C. Prat-Vidal C. Díaz-Güemes I. Crisóstomo V. Soler-Botija C. Roura S. (2014). Comparison of two preclinical myocardial infarct models: coronary coil deployment versus surgical ligation. *J. Transl. Med.* 12:137. 10.1186/1479-5876-12-137 24885652 PMC4047266

[B33] Gandolfi F. Vanelli A. Pennarossa G. Rahaman M. Acocella F. Brevini T. A. L. (2011). Large animal models for cardiac stem cell therapies. *Theriogenology* 75 1416–1425. 10.1016/j.theriogenology.2011.01.026 21463721

[B34] Gao L. R. Pei X. T. Ding Q. A. Chen Y. Zhang N. K. Chen H. Y. (2013). A critical challenge: dosage-related efficacy and acute complication intracoronary injection of autologous bone marrow mesenchymal stem cells in acute myocardial infarction. *Int. J. Cardiol.* 168 3191–3199. 10.1016/j.ijcard.2013.04.112 23651816

[B35] Gao M. Xin G. Qiu X. Wang Y. Liu G. (2016). Establishment of a rat model with diet-induced coronary atherosclerosis. *J. Biomed. Res.* 31 47–55. 28808185 10.7555/JBR.31.20160020 PMC5274512

[B36] Garcia-Dorado D. Théroux P. Elizaga J. Galiñanes M. Solares J. Riesgo M. (1987). Myocardial reperfusion in the pig heart model: infarct size and duration of coronary occlusion. *Cardiovasc. Res.* 21 537–544. 10.1093/cvr/21.7.537 3677143

[B37] Gargiulo S. Greco A. Gramanzini M. Petretta M. P. Ferro A. Larobina M. (2012). PET/CT imaging in mouse models of myocardial ischemia. *J. Biomed. Biotechnol.* 2012:541872. 22505813 10.1155/2012/541872 PMC3312322

[B38] Getz G. S. Reardon C. A. (2012). Animal models of atherosclerosis. *Arterioscler. Thromb. Vasc. Biol.* 32 1104–1115. 22383700 10.1161/ATVBAHA.111.237693 PMC3331926

[B39] Ghugre N. R. Pop M. Barry J. Connelly K. A. Wright G. A. (2013). Quantitative magnetic resonance imaging can distinguish remodeling mechanisms after acute myocardial infarction based on the severity of ischemic insult. *Magn. Reson. Med.* 70 1095–1105. 10.1002/mrm.24531 23165643

[B40] Giustino G. Dangas G. D. (2017). Ischemia-reperfusion injury and ischemic post-conditioning in acute myocardial infarction: Lost in translation. *Catheter. Cardiovasc. Interv.* 90 1068–1069. 10.1002/ccd.27436 29226578

[B41] Graham G. (2015). Disparities in cardiovascular disease risk in the United States. *Curr. Cardiol. Rev.* 11 238–245. 10.2174/1573403x11666141122220003 25418513 PMC4558355

[B42] Graham G. (2016). Racial and ethnic differences in acute coronary syndrome and myocardial infarction within the united states: from demographics to outcomes. *Clin. Cardiol.* 39 299–306. 10.1002/clc.22524 27028198 PMC6490862

[B43] Guo R. Morimatsu M. Feng T. Lan F. Chang D. Wan F. (2020). Stem cell-derived cell sheet transplantation for heart tissue repair in myocardial infarction. *Stem Cell Res. Ther.* 11:19. 31915074 10.1186/s13287-019-1536-y PMC6950817

[B44] Halim S. A. S. A. Ghafar N. A. Jubri Z. Das S. (2018). Induction of myocardial infarction in experimental animals: A review. *JCDR* 2018:12221.

[B45] Halkos M. E. Zhao Z.-Q. Kerendi F. Wang N.-P. Jiang R. Schmarkey L. S. (2008). Intravenous infusion of mesenchymal stem cells enhances regional perfusion and improves ventricular function in a porcine model of myocardial infarction. *Basic Res. Cardiol.* 103 525–536. 10.1007/s00395-008-0741-0 18704259

[B46] Haller C. Sobolewska B. Schibilsky D. Avci-Adali M. Schlensak C. Wendel H.-P. (2015). One-staged aptamer-based isolation and application of endothelial progenitor cells in a porcine myocardial infarction model. *Nucleic Acid Ther.* 25 20–26. 10.1089/nat.2014.0499 25494449

[B47] Hanes D. W. Wong M. L. Jenny Chang C. W. Humphrey S. Grayson J. K. Boyd W. D. (2015). Embolization of the first diagonal branch of the left anterior descending coronary artery as a porcine model of chronic trans-mural myocardial infarction. *J. Transl. Med.* 13:187. 26047812 10.1186/s12967-015-0547-4 PMC4634919

[B48] Harken A. H. Simson M. B. Haselgrove J. Wetstein L. Harden W. R. Barlow C. H. (1981). Early ischemia after complete coronary ligation in the rabbit, dog, pig, and monkey. *Am. J. Physiol.* 241 H202–H210. 7270707 10.1152/ajpheart.1981.241.2.H202

[B49] Hausenloy D. J. Yellon D. M. (2013). Myocardial ischemia-reperfusion injury: a neglected therapeutic target. *J. Clin. Invest.* 123 92–100. 10.1172/jci62874 23281415 PMC3533275

[B50] Hausenloy D. J. Yellon D. M. (2016). Ischaemic conditioning and reperfusion injury. *Nat. Rev. Cardiol.* 13 193–209. 10.1038/nrcardio.2016.5 26843289

[B51] Hausenloy D. J. Baxter G. Bell R. Bøtker H. E. Davidson S. M. Downey J. (2010). Translating novel strategies for cardioprotection: the Hatter Workshop Recommendations. *Basic Res. Cardiol.* 105 677–686. 10.1007/s00395-010-0121-4 20865418 PMC2965360

[B52] Hearse D. (2000). The elusive coypu: the importance of collateral flow and the search for an alternative to the dog. *Cardiovasc. Res.* 45 215–219. 10.1016/s0008-6363(99)00331-4

[B53] Henderson V. C. Kimmelman J. Fergusson D. Grimshaw J. M. Hackam D. G. (2013). Threats to validity in the design and conduct of preclinical efficacy studies: a systematic review of guidelines for in vivo animal experiments. *PLoS Med.* 10:e1001489. 10.1371/journal.pmed.1001489 23935460 PMC3720257

[B54] Heusch G. (2013). Cardioprotection: chances and challenges of its translation to the clinic. *Lancet* 381 166–175. 10.1016/s0140-6736(12)60916-7 23095318

[B55] Heusch G. (2015). Molecular basis of cardioprotection: signal transduction in ischemic pre-, post-, and remote conditioning. *Circ. Res.* 116 674–699. 10.1161/circresaha.116.305348 25677517

[B56] Heusch G. (2017). Critical issues for the translation of cardioprotection. *Circ. Res.* 120 1477–1486. 10.1161/circresaha.117.310820 28450365

[B57] Hill A. J. Iaizzo P. A. (2009). “Comparative Cardiac Anatomy,” in *Handbook of cardiac anatomy, physiology, and devices*, ed. Iaizzo P. A. (Totowa, NJ: Humana Press), 87–108. 10.1007/978-1-60327-372-5_6

[B58] Hirano A. Fujita J. Kanazawa H. Kawaguchi S. Handa N. Yamada Y. (2017). Cryoinjury-induced acute myocardial infarction model and ameroid constrictor-induced ischemic heart disease model in adult micro-mini pigs for preclinical studies. *Transl. Med. Commun.* 2:1. 10.1155/2014/571076 24817898 PMC4003740

[B59] Hirst A. Altman D. G. (2012). Are peer reviewers encouraged to use reporting guidelines? A survey of 116 health research journals. *PLoS One* 7:e35621. 10.1371/journal.pone.0035621.g001 22558178 PMC3338712

[B60] Hirst J. A. Howick J. Aronson J. K. Roberts N. Perera R. Koshiaris C. (2014). The need for randomization in animal trials: an overview of systematic reviews. *PLoS One* 9:e98856. 10.1371/journal.pone.0098856 24906117 PMC4048216

[B61] Hooijmans C. R. Ritskes-Hoitinga M. (2013). Progress in using systematic reviews of animal studies to improve translational research. *PLoS Med.* 10:e1001482. 10.1371/journal.pmed.1001482 23874162 PMC3712909

[B62] Hooijmans C. R. Leenaars M. Ritskes-Hoitinga M. A. (2010). gold standard publication checklist to improve the quality of animal studies, to fully integrate the Three Rs, and to make systematic reviews more feasible. *Altern. Lab. Anim.* 38 167–182. 10.1177/026119291003800208 20507187

[B63] Houtgraaf J. H. de Jong R. Kazemi K. de Groot D. van der Spoel T. I. G. Arslan F. (2013). Intracoronary infusion of allogeneic mesenchymal precursor cells directly after experimental acute myocardial infarction reduces infarct size, abrogates adverse remodeling, and improves cardiac function. *Circ. Res.* 113 153–166. 10.1161/circresaha.112.300730 23658436

[B64] Ingber D. E. (2018). Developmentally inspired human “organs on chips”. *Development* 145:16. 10.1242/dev.156125 PMC6124544 29776965

[B65] Ingber D. E. (2020). Is it time for reviewer 3 to request human organ chip experiments instead of animal validation studies? *Adv. Sci.* 7:2002030. 10.1002/advs.202002030 33240763 PMC7675190

[B66] Ioannidis J. P. A. (2005). Why most published research findings are false. *PLoS Med.* 2:e124. 10.1371/journal.pmed.0020124 16060722 PMC1182327

[B67] Ioannidis J. P. A. (2016). Why most clinical research is not useful. *PLoS Med.* 13:e1002049. 10.1371/journal.pmed.1002049 27328301 PMC4915619

[B68] Ishida M. Miyagawa S. Saito A. Fukushima S. Harada A. Ito E. (2019). Transplantation of Human-induced Pluripotent Stem Cell-derived Cardiomyocytes Is Superior to Somatic Stem Cell Therapy for Restoring Cardiac Function and Oxygen Consumption in a Porcine Model of Myocardial Infarction. *Transplantation* 103 291–298. 10.1097/tp.0000000000002384 30119058 PMC6365242

[B69] Janssens S. Dubois C. Bogaert J. Theunissen K. Deroose C. Desmet W. (2006). Autologous bone marrow-derived stem-cell transfer in patients with ST-segment elevation myocardial infarction: double-blind, randomised controlled trial. *Lancet* 367 113–121. 10.1016/s0140-6736(05)67861-0 16413875

[B70] Joudinaud T. M. Kegel C. L. Gabster A. A. Sanz M. L. MacDonald A. Propp D. (2005). An experimental method for the percutaneous induction of a posterolateral infarct and functional ischemic mitral regurgitation. *J. Heart Valve Dis.* 14 460–466. 16116871

[B71] Kamimura R. Suzuki S. Nozaki S. Sakamoto H. Maruno H. Kawaida H. (1996). Branching patterns in coronary artery and ischemic areas induced by coronary arterial occlusion in the CLAWN miniature pig. *Exp. Anim.* 45 149–153. 10.1538/expanim.45.149 8726139

[B72] Khan M. A. (1984). Minipig: advantages and disadvantages as a model in toxicity testing. *J. Am. Coll Toxicol.* 3 337–342. 10.3109/10915818409104396

[B73] Kim J.-H. Chung H.-S. Antonisamy P. Lee S. R. Bae H. (2014). Cardioprotective effect of rhizomes of Acorus gramineus against isoproterenol-induced cardiac damage in pigs. *Cardiovasc. Toxicol.* 14 183–192. 10.1007/s12012-014-9243-5 24420420

[B74] Kim M. C. Kim Y. S. Kang W. S. Lee K. H. Cho M. Hong M. H. (2017). Intramyocardial injection of stem cells in pig myocardial infarction model: the first trial in korea. *J. Korean. Med. Sci.* 32 1708–1712. 10.3346/jkms.2017.32.10.1708 28875618 PMC5592188

[B75] Kloner R. A. (2013). Current state of clinical translation of cardioprotective agents for acute myocardial infarction. *Circ. Res.* 113 451–463. 10.1161/circresaha.112.300627 23908332

[B76] Kloner R. A. Rezkalla S. H. (2004). Cardiac protection during acute myocardial infarction: where do we stand in 2004? *J. Am. Coll. Cardiol.* 44 276–286. 10.1016/j.jacc.2004.03.068 15261919

[B77] Kraitchman D. L. Heldman A. W. Atalar E. Amado L. C. Martin B. J. Pittenger M. F. (2003). In vivo magnetic resonance imaging of mesenchymal stem cells in myocardial infarction. *Circulation* 107 2290–2293. 10.1161/01.cir.0000070931.62772.4e 12732608

[B78] Krause U. Harter C. Seckinger A. Wolf D. Reinhard A. Bea F. (2007). Intravenous delivery of autologous mesenchymal stem cells limits infarct size and improves left ventricular function in the infarcted porcine heart. *Stem Cells Dev.* 16 31–37. 10.1089/scd.2006.0089 17348804

[B79] Krueger M. A. Huke S. S. Glenny R. W. (2013). Visualizing regional myocardial blood flow in the mouse. *Circ. Res.* 112 e88–e97. 23513055 10.1161/CIRCRESAHA.113.301162

[B80] Kumar D. Hacker T. A. Buck J. Whitesell L. F. Kaji E. H. Douglas P. S. (2005). Distinct mouse coronary anatomy and myocardial infarction consequent to ligation. *Coron. Artery Dis.* 16 41–44. 10.1097/00019501-200502000-00008 15654199

[B81] Laflamme M. A. Chen K. Y. Naumova A. V. Muskheli V. Fugate J. A. Dupras S. K. (2007). Cardiomyocytes derived from human embryonic stem cells in pro-survival factors enhance function of infarcted rat hearts. *Nat. Biotechnol.* 25 1015–1024. 10.1038/nbt1327 17721512

[B82] Lee Y. T. Lin H. Y. Chan Y. W. F. Li K. H. C. To O. T. L. Yan B. P. (2017). Mouse models of atherosclerosis: a historical perspective and recent advances. *Lipids Health Dis.* 16:12. 28095860 10.1186/s12944-016-0402-5 PMC5240327

[B83] Lelovas P. P. Kostomitsopoulos N. G. Xanthos T. T. A. (2014). comparative anatomic and physiologic overview of the porcine heart. *J. Am. Assoc. Lab. Anim. Sci.* 53 432–438. 25255064 PMC4181683

[B84] Li R. K. Weisel R. D. Mickle D. A. Jia Z. Q. Kim E. J. Sakai T. (2000). Autologous porcine heart cell transplantation improved heart function after a myocardial infarction. *J. Thorac. Cardiovasc. Surg.* 119 62–68. 10.1016/s0022-5223(00)70218-2 10612762

[B85] Li X. Zhang F. Song G. Gu W. Chen M. Yang B. (2013). Intramyocardial injection of pig pluripotent stem cells improves left ventricular function and perfusion: A study in a porcine model of acute myocardial infarction. *PLoS One.* 8:e66688. 10.1371/journal.pone.0066688 23805264 PMC3689724

[B86] Lim K. H. Cho J. Y. Kim B. Bae B.-S. Kim J.-H. (2014). Red ginseng (Panax ginseng) decreases isoproterenol-induced cardiac injury via antioxidant properties in porcine. *J. Med. Food.* 17 111–118. 10.1089/jmf.2013.2768 24456361 PMC3901382

[B87] Lim M. Wang W. Liang L. Han Z.-B. Li Z. Geng J. (2018). Intravenous injection of allogeneic umbilical cord-derived multipotent mesenchymal stromal cells reduces the infarct area and ameliorates cardiac function in a porcine model of acute myocardial infarction. *Stem Cell Res. Ther.* 9:129. 29751831 10.1186/s13287-018-0888-z PMC5948807

[B88] Liu X. Wang Y. Tang M. Liu Y. Hu L. Gu Y. (2020). Three-dimensional visualization of coronary microvasculature in rats with myocardial infarction. *Microvasc. Res.* 130:103990. 10.1016/j.mvr.2020.103990 32088162

[B89] Locatelli P. Olea F. D. Mendiz O. Salmo F. Fazzi L. Hnatiuk A. (2011). An ovine model of postinfarction dilated cardiomyopathy in animals with highly variable coronary anatomy. *ILAR J.* 52 E16–E21. 21454923 10.1093/ilar.52.1.e16

[B90] Locher M. R. Razumova M. V. Stelzer J. E. Norman H. S. Patel J. R. Moss R. L. (2009). Determination of rate constants for turnover of myosin isoforms in rat myocardium: implications for in vivo contractile kinetics. *Am. J. Physiol. Heart Circ. Physiol.* 297 H247–H256. 19395549 10.1152/ajpheart.00922.2008 PMC2711735

[B91] Lu M. Zhao S. Liu Q. Jiang S. Song P. Qian H. (2012). Transplantation with autologous mesenchymal stem cells after acute myocardial infarction evaluated by magnetic resonance imaging: an experimental study. *J. Thorac. Imaging* 27 125–135. 10.1097/rti.0b013e31820446fa 21336180

[B92] Ludman A. J. Yellon D. M. Hausenloy D. J. (2010). Cardiac preconditioning for ischaemia: lost in translation. *Dis. Model Mech.* 3 35–38. 10.1242/dmm.003855 20075380

[B93] Lunde K. Solheim S. Aakhus S. Arnesen H. Abdelnoor M. Egeland T. (2006). Intracoronary injection of mononuclear bone marrow cells in acute myocardial infarction. *N. Engl. J. Med.* 355 1199–1209. 16990383 10.1056/NEJMoa055706

[B94] Lüscher T. F. (2019). Back to square one. *Eur. Heart J.* 40 1031–1033. 30933286 10.1093/eurheartj/ehz094

[B95] Makkar R. R. Price M. J. Lill M. Frantzen M. Takizawa K. Kleisli T. (2005). Intramyocardial injection of allogenic bone marrow-derived mesenchymal stem cells without immunosuppression preserves cardiac function in a porcine model of myocardial infarction. *J. Cardiovasc. Pharmacol. Ther.* 10 225–233. 10.1177/107424840501000403 16382259

[B96] Makkar R. R. Smith R. R. Cheng K. Malliaras K. Thomson L. E. Berman D. (2012). Intracoronary cardiosphere-derived cells for heart regeneration after myocardial infarction (CADUCEUS): a prospective, randomised phase 1 trial. *Lancet* 379 895–904. 10.1016/s0140-6736(12)60195-0 22336189 PMC4326004

[B97] Maxwell M. P. Hearse D. J. Yellon D. M. (1987). Species variation in the coronary collateral circulation during regional myocardial ischaemia: a critical determinant of the rate of evolution and extent of myocardial infarction. *Cardiovasc. Res.* 21 737–746. 10.1093/cvr/21.10.737 3440266

[B98] Menasché P. (2018). Cell therapy trials for heart regeneration - lessons learned and future directions. *Nat. Rev. Cardiol.* 15 659–671. 10.1038/s41569-018-0013-0 29743563

[B99] Milani-Nejad N. Janssen P. M. L. (2014). Small and large animal models in cardiac contraction research: advantages and disadvantages. *Pharmacol. Ther.* 141 235–249. 10.1016/j.pharmthera.2013.10.007 24140081 PMC3947198

[B100] Miura T. Miki T. (2008). Limitation of myocardial infarct size in the clinical setting: current status and challenges in translating animal experiments into clinical therapy. *Basic Res. Cardiol.* 103 501–513. 10.1007/s00395-008-0743-y 18716709

[B101] Miyagawa S. Domae K. Yoshikawa Y. Fukushima S. Nakamura T. Saito A. (2017). Phase I Clinical Trial of Autologous Stem Cell-Sheet Transplantation Therapy for Treating Cardiomyopathy. *J Am. Heart Assoc.* 6:4. 10.1161/JAHA.116.003918 PMC5532985 28381469

[B102] Morrissey P. J. Murphy K. R. Daley J. M. Schofield L. Turan N. N. Arunachalam K. (2017). A novel method of standardized myocardial infarction in aged rabbits. *Am. J. Physiol. Heart Circ. Physiol.* 312 H959–H967. 28213402 10.1152/ajpheart.00582.2016 PMC5451580

[B103] Mu D. Zhang X.-L. Xie J. Yuan H.-H. Wang K. Huang W. (2016). Intracoronary Transplantation of Mesenchymal Stem Cells with Overexpressed Integrin-Linked Kinase Improves Cardiac Function in Porcine Myocardial Infarction. *Sci. Rep.* 6:19155. 26750752 10.1038/srep19155 PMC4707493

[B104] Muller C. A. Opie L. H. Hamm C. W. Peisach M. Pineda C. A. Thandroyen F. T. (1988). Verapamil and tiapamil in prevention of ventricular fibrillation in pigs with coronary ligation. Comparative effects on left ventricular function. *Circulation* 78 227–232. 10.1161/01.cir.78.1.227 3383406

[B105] National Research Council (Us) Committee on Scientific and Humane Issues in the Use of Random Source Dogs and Cats in Research (2009). *Use of Dogs and Cats in Research: Public Perception and Evolution of Laws and Guidelines - Scientific and Humane Issues in the Use of Random Source Dogs and Cats in Research - NCBI Bookshelf.* Washington, DC: National Research Council.

[B106] Nguyen P. K. Wu J. C. (2015). Large animal models of ischemic cardiomyopathy: are they enough to bridge the translational gap? *J. Nucl. Cardiol.* 22 666–672. 10.1007/s12350-015-0078-7 25777782

[B107] Nicolini F. Gherli T. (2009). Alternatives to transplantation in the surgical therapy for heart failure. *Eur. J. Cardiothorac. Surg.* 35 214–228. 10.1016/j.ejcts.2008.11.003 19091591

[B108] Anon J. (2013). Announcement: Reducing our irreproducibility. *Nature* 496 398–398. 10.1038/496398a

[B109] Nunoya T. Shibuya K. Saitoh T. Yazawa H. Nakamura K. Baba Y. (2007). Use of Miniature Pig for Biomedical Research, with Reference to Toxicologic Studies. *J. Toxicol. Pathol.* 20 125–132. 10.1293/tox.20.125

[B110] Okura H. Saga A. Soeda M. Miyagawa S. Sawa Y. Daimon T. (2012). Intracoronary artery transplantation of cardiomyoblast-like cells from human adipose tissue-derived multi-lineage progenitor cells improve left ventricular dysfunction and survival in a swine model of chronic myocardial infarction. *Biochem. Biophys. Res. Commun.* 425 859–865. 10.1016/j.bbrc.2012.08.004 22898045

[B111] Penicka M. Horak J. Kobylka P. Pytlik R. Kozak T. Belohlavek O. (2007). Intracoronary injection of autologous bone marrow-derived mononuclear cells in patients with large anterior acute myocardial infarction: a prematurely terminated randomized study. *J. Am. Coll. Cardiol.* 49 2373–2374. 10.1016/j.jacc.2007.04.009 17572255

[B112] Perel P. Roberts I. Sena E. Wheble P. Briscoe C. Sandercock P. (2007). Comparison of treatment effects between animal experiments and clinical trials: systematic review. *BMJ* 334:197. 10.1136/bmj.39048.407928.be 17175568 PMC1781970

[B113] Perin E. C. Willerson J. T. Pepine C. J. Henry T. D. Ellis S. G. Zhao D. X. M. (2012). Effect of transendocardial delivery of autologous bone marrow mononuclear cells on functional capacity, left ventricular function, and perfusion in chronic heart failure: the FOCUS-CCTRN trial. *JAMA* 307 1717–1726. 22447880 10.1001/jama.2012.418 PMC3600947

[B114] Podesser B. Wollenek G. Seitelberger R. Siegel H. Wolner E. Firbas W. (1997). Epicardial branches of the coronary arteries and their distribution in the rabbit heart: The rabbit heart as a model of regional ischemia. *Anatomical. Record* 1997:1. 10.1002/(SICI)1097-0185(199704)247:4<521::AID-AR11>3.0.CO;2-R 9096792

[B115] Potz B. A. Scrimgeour L. A. Pavlov V. I. Sodha N. R. Abid M. R. Sellke F. W. (2018). Extracellular vesicle injection improves myocardial function and increases angiogenesis in a swine model of chronic ischemia. *J. Am. Heart Assoc.* 7:12. 10.1161/JAHA.117.008344 PMC6220556 29895586

[B116] Pound P. Ritskes-Hoitinga M. (2018). Is it possible to overcome issues of external validity in preclinical animal research? Why most animal models are bound to fail. *J. Transl. Med.* 16:304. 30404629 10.1186/s12967-018-1678-1 PMC6223056

[B117] Pound P. Ebrahim S. Sandercock P. Bracken M. B. Roberts I. (2004). Reviewing Animal Trials Systematically (RATS) Group. Where is the evidence that animal research benefits humans? *BMJ* 328 514–517. 10.1136/bmj.328.7438.514 14988196 PMC351856

[B118] Price M. J. Chou C.-C. Frantzen M. Miyamoto T. Kar S. Lee S. (2006). Intravenous mesenchymal stem cell therapy early after reperfused acute myocardial infarction improves left ventricular function and alters electrophysiologic properties. *Int. J. Cardiol.* 111 231–239. 10.1016/j.ijcard.2005.07.036 16246440

[B119] Principles and Guidelines for Reporting Preclinical Research and National Institutes of Health (NIH) (2021). Available online at: https://www.nih.gov/research-training/rigor-reproducibility/principles-guidelines-reporting-preclinical-research (acceessed date 28, March 2021)

[B120] Quintana H. K. Janszky I. Kanar A. Gigante B. Druid H. Ahlbom A. (2018). Comorbidities in relation to fatality of first myocardial infarction. *Cardiovasc. Pathol.* 32 32–37. 10.1016/j.carpath.2017.11.002 29175662

[B121] Quyyumi A. A. Vasquez A. Kereiakes D. J. Klapholz M. Schaer G. L. Abdel-Latif A. (2017). PreSERVE-AMI: A Randomized, Double-Blind, Placebo-Controlled Clinical Trial of Intracoronary Administration of Autologous CD34+ Cells in Patients With Left Ventricular Dysfunction Post STEMI. *Circ. Res.* 120 324–331. 10.1161/circresaha.115.308165 27821724 PMC5903285

[B122] Rabbani S. Ahmadi H. Fayazzadeh E. Sahebjam M. Boroumand M. A. Sotudeh M. (2008). Development of an ovine model of myocardial infarction. *ANZ J. Surg.* 78 78–81. 10.1111/j.1445-2197.2007.04359.x 18199212

[B123] Rabbani S. Soleimani M. Sahebjam M. Imani M. Nassiri S. M. Atashi A. (2017). Effects of endothelial and mesenchymal stem cells on improving myocardial function in a sheep animal model. *J. Tehran. Heart Cent.* 12 65–71. 28828021 PMC5558057

[B124] Ramirez F. D. Motazedian P. Jung R. G. Di Santo P. MacDonald Z. D. Moreland R. (2017). Methodological rigor in preclinical cardiovascular studies: targets to enhance reproducibility and promote research translation. *Circ. Res.* 120 1916–1926. 10.1161/circresaha.117.310628 28373349 PMC5466021

[B125] Ranasinghe I. Shojaee A. Bikdeli B. Gupta A. Chen R. Ross J. S. (2015). Poorly cited articles in peer-reviewed cardiovascular journals from 1997 to 2007: analysis of 5-year citation rates. *Circulation* 131 1755–1762. 10.1161/circulationaha.114.015080 25812573 PMC4560203

[B126] Ribas J. Sadeghi H. Manbachi A. Leijten J. Brinegar K. Zhang Y. S. (2016). Cardiovascular Organ-on-a-Chip Platforms for Drug Discovery and Development. *Appl. Vitro Toxicol.* 2 82–96. 10.1089/aivt.2016.0002 28971113 PMC5044977

[B127] Ribitsch I. Baptista P. M. Lange-Consiglio A. Melotti L. Patruno M. Jenner F. (2020). Large animal models in regenerative medicine and tissue engineering: to do or not to do. *Front. Bioeng. Biotechnol.* 8:972. 10.3389/fbioe.2020.00972 32903631 PMC7438731

[B128] Richards D. J. Li Y. Kerr C. M. Yao J. Beeson G. C. Coyle R. C. (2020). Human cardiac organoids for the modelling of myocardial infarction and drug cardiotoxicity. *Nat. Biomed. Eng.* 4 446–462. 10.1038/s41551-020-0539-4 32284552 PMC7422941

[B129] Richter S. H. Garner J. P. Würbel H. (2009). Environmental standardization: cure or cause of poor reproducibility in animal experiments? *Nat. Methods* 6 257–261. 10.1038/nmeth.1312 19333241

[B130] Riehle C. Bauersachs J. (2019). Small animal models of heart failure. *Cardiovasc. Res.* 115 1838–1849. 10.1093/cvr/cvz161 31243437 PMC6803815

[B131] Rienzo M. Imbault J. El Boustani Y. Beurton A. Carlos Sampedrano C. Pasdois P. (2020). A total closed chest sheep model of cardiogenic shock by percutaneous intracoronary ethanol injection. *Sci. Rep.* 10:12417. 32709984 10.1038/s41598-020-68571-5 PMC7381645

[B132] Romagnuolo R. Masoudpour H. Porta-Sánchez A. Qiang B. Barry J. Laskary A. (2019). Human Embryonic Stem Cell-Derived Cardiomyocytes Regenerate the Infarcted Pig Heart but Induce Ventricular Tachyarrhythmias. *Stem Cell Rep.* 12 967–981. 10.1016/j.stemcr.2019.04.005 31056479 PMC6524945

[B133] Sandercock P. Roberts I. (2002). Systematic reviews of animal experiments. *Lancet* 360:586. 10.1016/s0140-6736(02)09812-4 12241927

[B134] Savarese G. Lund L. H. (2017). Global public health burden of heart failure. *Card Fail Rev.* 3 7–11. 28785469 10.15420/cfr.2016:25:2 PMC5494150

[B135] Savoji H. Mohammadi M. H. Rafatian N. Toroghi M. K. Wang E. Y. Zhao Y. (2019). Cardiovascular disease models: A game changing paradigm in drug discovery and screening. *Biomaterials* 198 3–26. 10.1016/j.biomaterials.2018.09.036 30343824 PMC6397087

[B136] Schuleri K. H. Boyle A. J. Centola M. Amado L. C. Evers R. Zimmet J. M. (2008). The adult Göttingen minipig as a model for chronic heart failure after myocardial infarction: focus on cardiovascular imaging and regenerative therapies. *Comp. Med.* 58 568–579. 19149414 PMC2710749

[B137] Sharp T. E. Schena G. J. Hobby A. R. Starosta T. Berretta R. M. Wallner M. (2017). Cortical bone stem cell therapy preserves cardiac structure and function after myocardial infarction. *Circ. Res.* 121 1263–1278. 10.1161/circresaha.117.311174 28912121 PMC5681384

[B138] Shudo Y. Cohen J. E. Macarthur J. W. Atluri P. Hsiao P. F. Yang E. C. (2013). Spatially oriented, temporally sequential smooth muscle cell-endothelial progenitor cell bi-level cell sheet neovascularizes ischemic myocardium. *Circulation* 128 (11 Suppl. 1) S59–S68. 24030422 10.1161/CIRCULATIONAHA.112.000293 PMC4111240

[B139] Shudo Y. Miyagawa S. Fukushima S. Saito A. Shimizu T. Okano T. (2011). Novel regenerative therapy using cell-sheet covered with omentum flap delivers a huge number of cells in a porcine myocardial infarction model. *J. Thorac. Cardiovasc. Surg.* 142 1188–1196. 10.1016/j.jtcvs.2011.07.002 21924436

[B140] Shudo Y. Miyagawa S. Ohkura H. Fukushima S. Saito A. Shiozaki M. (2014). Addition of mesenchymal stem cells enhances the therapeutic effects of skeletal myoblast cell-sheet transplantation in a rat ischemic cardiomyopathy model. *Tissue Eng. Part A.* 20 728–739. 24164292 10.1089/ten.tea.2012.0534 PMC3926175

[B141] Sjaastad I. Grund F. Ilebekk A. (2000). Effects on infarct size and on arrhythmias by controlling reflow after myocardial ischaemia in pigs. *Acta Physiol. Scand.* 169 195–201. 10.1046/j.1365-201x.2000.00735.x 10886034

[B142] Spadaro J. Fishbein M. C. Hare C. Pfeffer M. A. Maroko P. R. (1980). Characterization of myocardial infarcts in the rat. *Arch. Pathol. Lab. Med.* 104 179–183. 6892678

[B143] Spannbauer A. Traxler D. Zlabinger K. Gugerell A. Winkler J. Mester-Tonczar J. (2019). Large animal models of heart failure with reduced ejection fraction (hfref). *Front. Cardiovasc. Med.* 6:117. 10.3389/fcvm.2019.00117 31475161 PMC6702665

[B144] Steele A. N. MacArthur J. W. Woo Y. J. (2017). Stem cell therapy: healing or hype? why stem cell delivery doesn’t work. *Circ. Res.* 120 1868–1870. 10.1161/circresaha.117.310584 28596172 PMC5947316

[B145] Stricker-Krongrad A. Shoemake C. Brocksmith D. Liu J. Hamlin R. Bouchard G. (2017). Comparative cardiovascular physiology and pathology in selected lineages of minipigs. *Toxicol. Res. Appl.* 1:239784731769636. 10.1177/2397847317696367

[B146] Stubhan M. Markert M. Mayer K. Trautmann T. Klumpp A. Henke J. (2008). Evaluation of cardiovascular and ECG parameters in the normal, freely moving Göttingen Minipig. *J. Pharmacol. Toxicol. Methods* 57 202–211. 10.1016/j.vascn.2008.02.001 18434212

[B147] Sun S. Jiang Y. Zhen Z. Lai W.-H. Liao S. Tse H.-F. (2020). Establishing a Swine Model of Post-myocardial Infarction Heart Failure for Stem Cell Treatment. *J. Vis. Exp.* 2020:159. 10.3791/60392 32510509

[B148] Suzuki G. Young R. F. Leiker M. M. Suzuki T. (2016). Heart-Derived Stem Cells in Miniature Swine with Coronary Microembolization: Novel Ischemic Cardiomyopathy Model to Assess the Efficacy of Cell-Based Therapy. *Stem Cells Int.* 2016: 6940195. 10.1155/2016/6940195 PMC5055979 27738436

[B149] Tang Y.-P. Liu Y. Fan Y.-J. Zhao Y.-Y. Feng J.-Q. Liu Y. (2018). To develop a novel animal model of myocardial infarction: A research imperative. *Anim. Models Exp. Med.* 1 36–39. 10.1002/ame2.12010 30891545 PMC6357429

[B150] Techiryan G. Weil B. R. Palka B. A. Canty J. M. (2018). Effect of intracoronary metformin on myocardial infarct size in swine. *Circ. Res.* 123 986–995. 10.1161/circresaha.118.313341 30355033 PMC6207210

[B151] Thomas D. Ferrari V. A. Janik M. Kim D. H. Pickup S. Glickson J. D. (2004). Quantitative assessment of regional myocardial function in a rat model of myocardial infarction using tagged MRI. *MAGMA* 17 179–187. 10.1007/s10334-004-0051-y 15517473 PMC2964083

[B152] Thomas R. Thai K. Barry J. Wright G. A. Strauss B. H. Ghugre N. R. (2021). T2-based area-at-risk and edema are influenced by ischemic duration in acute myocardial infarction. *Magn. Reson. Imaging* 79 1–4. 10.1016/j.mri.2021.02.011 33652063

[B153] Timmers L. Lim S. K. Hoefer I. E. Arslan F. Lai R. C. van Oorschot A. A. M. (2011). Human mesenchymal stem cell-conditioned medium improves cardiac function following myocardial infarction. *Stem Cell Res.* 6 206–214. 21419744 10.1016/j.scr.2011.01.001

[B154] Tohyama S. Kobayashi E. (2019). Age-Appropriateness of Porcine Models Used for Cell Transplantation. *Cell Trans.* 28 224–228. 10.1177/0963689718817477 PMC6362526 30525991

[B155] Trankle C. Thurber C. J. Toldo S. Abbate A. (2016). Mitochondrial membrane permeability inhibitors in acute myocardial infarction: still awaiting translation. *JACC Basic Transl. Sci.* 1 524–535. 30167535 10.1016/j.jacbts.2016.06.012 PMC6113419

[B156] Ungerleider J. L. Christman K. L. (2014). Concise review: injectable biomaterials for the treatment of myocardial infarction and peripheral artery disease: translational challenges and progress. *Stem Cells Transl. Med.* 3 1090–1099. 25015641 10.5966/sctm.2014-0049 PMC4149304

[B157] van der Velden J. Merkus D. Klarenbeek B. R. James A. T. Boontje N. M. Dekkers D. H. W. (2004). Alterations in myofilament function contribute to left ventricular dysfunction in pigs early after myocardial infarction. *Circ. Res.* 95 e85–e95. 15528471 10.1161/01.RES.0000149531.02904.09

[B158] van der Worp H. B. Howells D. W. Sena E. S. Porritt M. J. Rewell S. O’Collins V. (2010). Can animal models of disease reliably inform human studies? *PLoS Med.* 7:e1000245. 10.1371/journal.pmed.1000245 20361020 PMC2846855

[B159] van Luijk J. Bakker B. Rovers M. M. Ritskes-Hoitinga M. de Vries R. B. M. Leenaars M. (2014). Systematic reviews of animal studies; missing link in translational research? *PLoS One* 9:e89981. 10.1371/journal.pone.0089981 24670965 PMC3966727

[B160] Virani S. S. Alonso A. Aparicio H. J. Benjamin E. J. Bittencourt M. S. Callaway C. W. (2021). Heart Disease and Stroke Statistics-2021 Update: A Report From the American Heart Association. *Circulation* 143 e254–e743. 33501848 10.1161/CIR.0000000000000950 PMC13036842

[B161] Voelkl B. Vogt L. Sena E. S. Würbel H. (2018). Reproducibility of preclinical animal research improves with heterogeneity of study samples. *PLoS Biol.* 16:e2003693. 10.1371/journal.pbio.2003693 29470495 PMC5823461

[B162] Wang B. Zhang L. Cao H. Yang J. Wu M. Ma Y. (2017). Myoblast transplantation improves cardiac function after myocardial infarction through attenuating inflammatory responses. *Oncotarget* 8 68780–68794. 28978156 10.18632/oncotarget.18244 PMC5620296

[B163] Wang L. Tao T. Su W. Yu H. Yu Y. Qin J. A. (2017). disease model of diabetic nephropathy in a glomerulus-on-a-chip microdevice. *Lab. Chip.* 17 1749–1760. 28418422 10.1039/c7lc00134g

[B164] Wang X. Jameel M. N. Li Q. Mansoor A. Qiang X. Swingen C. (2009). Stem cells for myocardial repair with use of a transarterial catheter. *Circulation* 120 (11 Suppl.) S238–S246. 19752374 10.1161/CIRCULATIONAHA.109.885236 PMC3977692

[B165] Watanabe E. Smith D. M. Delcarpio J. B. Sun J. Smart F. W. Van Meter C. H. (1998). Cardiomyocyte transplantation in a porcine myocardial infarction model. *Cell Trans.* 7 239–246. 10.1177/096368979800700302 9647433

[B166] Weaver M. E. Pantely G. A. Bristow J. D. Ladley H. D. A. (1986). quantitative study of the anatomy and distribution of coronary arteries in swine in comparison with other animals and man. *Cardiovasc. Res.* 20 907–917. 3802126 10.1093/cvr/20.12.907

[B167] Wolf D. Reinhard A. Seckinger A. Katus H. A. Kuecherer H. Hansen A. (2009). Dose-dependent effects of intravenous allogeneic mesenchymal stem cells in the infarcted porcine heart. *Stem Cells Dev.* 18 321–329. 18435573 10.1089/scd.2008.0019

[B168] Wollert K. C. Meyer G. P. Müller-Ehmsen J. Tschöpe C. Bonarjee V. Larsen A. I. (2017). Intracoronary autologous bone marrow cell transfer after myocardial infarction: the BOOST-2 randomised placebo-controlled clinical trial. *Eur. Heart J.* 38 2936–2943. 28431003 10.1093/eurheartj/ehx188

[B169] Wu Q. Liu J. Wang X. Feng L. Wu J. Zhu X. (2020). Organ-on-a-chip: recent breakthroughs and future prospects. *Biomed. Eng. Online* 19:9. 32050989 10.1186/s12938-020-0752-0 PMC7017614

[B170] Yang Y. Gruwel M. L. Dreessen de Gervai P. Sun J. Jilkina O. Gussakovsky E. (2012). MRI study of cryoinjury infarction in pig hearts: i. Effects of intrapericardial delivery of bFGF/VEGF embedded in alginate beads. *NMR Biomed.* 25 177–188. 21960023 10.1002/nbm.1736

[B171] Yang Y. Sun J. Gervai P. Gruwel M. L. Jilkina O. Gussakovsky E. (2010). Characterization of cryoinjury-induced infarction with manganese-and gadolinium-enhanced MRI and optical spectroscopy in pig hearts. *Magn. Reson. Imag.* 28 753–766. 10.1016/j.mri.2010.02.001 20395099

[B172] Yellon D. M. Hausenloy D. J. (2007). Myocardial reperfusion injury. *N. Engl. J. Med.* 357 1121–1135. 17855673 10.1056/NEJMra071667

[B173] Zhao J.-J. Liu X.-C. Kong F. Qi T.-G. Cheng G.-H. Wang J. (2014). Bone marrow mesenchymal stem cells improve myocardial function in a swine model of acute myocardial infarction. *Mol. Med. Rep.* 10 1448–1454. 25060678 10.3892/mmr.2014.2378

